# Microbiota-Mitochondria Inter-Talk: A Potential Therapeutic Strategy in Obesity and Type 2 Diabetes

**DOI:** 10.3390/antiox9090848

**Published:** 2020-09-10

**Authors:** Teresa Vezza, Zaida Abad-Jiménez, Miguel Marti-Cabrera, Milagros Rocha, Víctor Manuel Víctor

**Affiliations:** 1 Service of Endocrinology and Nutrition, University Hospital Doctor Peset, Foundation for the Promotion of Health and Biomedical Research in the Valencian Region (FISABIO), 46017 Valencia, Spain; vezza_ter@gva.es (T.V.); zaiaji@alumni.uv.es (Z.A.-J.); 2 Department of Pharmacology, University of Valencia, 46010 Valencia, Spain; miguel.marti@uv.es; 3 CIBERehd—Department of Pharmacology, University of Valencia, 46010 Valencia, Spain; 4 Department of Physiology, University of Valencia, 46010 Valencia, Spain

**Keywords:** gut microbiota, inflammation, mitochondria, mitochondrial oxidative/nitrosative stress, obesity, type 2 diabetes

## Abstract

The rising prevalence of obesity and type 2 diabetes (T2D) is a growing concern worldwide. New discoveries in the field of metagenomics and clinical research have revealed that the gut microbiota plays a key role in these metabolic disorders. The mechanisms regulating microbiota composition are multifactorial and include resistance to stress, presence of pathogens, diet, cultural habits and general health conditions. Recent evidence has shed light on the influence of microbiota quality and diversity on mitochondrial functions. Of note, the gut microbiota has been shown to regulate crucial transcription factors, coactivators, as well as enzymes implicated in mitochondrial biogenesis and metabolism. Moreover, microbiota metabolites seem to interfere with mitochondrial oxidative/nitrosative stress and autophagosome formation, thus regulating the activation of the inflammasome and the production of inflammatory cytokines, key players in chronic metabolic disorders. This review focuses on the association between intestinal microbiota and mitochondrial function and examines the mechanisms that may be the key to their use as potential therapeutic strategies in obesity and T2D management.

## 1. Introduction

The worldwide prevalence of obesity and type 2 diabetes (T2D) has risen dramatically in the last decade, becoming a public health emergency. The World Health Organization [1] estimates that cases of obesity have tripled in 25 years, affecting children/adolescents (124 million in 2016) and adults (650 million in 2016) of both sexes. The global increase of the body mass index (BMI) was associated with 4.0 million deaths in 2015 [2], which has led to predictions that 300 million will be affected by obesity-related diseases by 2025 [3,4]. As with any other chronic disease, obesity and its associated comorbidities are related to a series of personal, financial, familial and social issues, as well as a higher mortality rate. Increased blood glucose and T2D, physical limitations and disabilities, musculoskeletal complications, certain psychiatric disorders, sexual dysfunction and cardiometabolic risk are some of the problems that negatively affect the lives of individuals with obesity [5]. In accordance with the WHO, overweight and obesity account for 44% of diabetes cases, 23% of ischemic heart disease patients and around 7–41% of certain cancers [6]. Of these diseases, T2D is most strongly associated with obesity, and the prevalence of obesity-related diabetes is expected to quadruple in a few years [7]. Consequently, obesity is currently the largest global chronic health problem, and its prevention and management are of vital importance. 

Obesity is a multifactorial disease involving a complex network of physical, systemic and physiological disorders. This condition develops as a consequence of an imbalance due to excessive intake and low expenditure of energy, leading to an abnormal accumulation of lipids in metabolic tissues, particularly adipose tissue and the liver [8,9]. Adipose tissue is mostly implicated in systemic metabolic homeostasis, acting as an autocrine and endocrine organ by releasing active mediators named adipokines. The dysregulation of these secretory factors, caused by excess adiposity and adipocyte dysfunction, can promote macrophage infiltration in the inflamed adipose tissue [10,11,12]. Accordingly, the accumulation of macrophages results in the secretion of different proinflammatory mediators, such as interleukin (IL)-6 and tumor necrosis factor (TNF)-α, which can potentially contribute to the initiation and progression of obesity-induced metabolic complications [9]. If the magnitude of cytokines secretion is large enough, these can leak out of the tissue, raising circulating levels and producing endocrine effects in distant organ systems (such as muscle and the liver), thus exacerbating the systemic insulin resistance and beta (β)-cell dysfunction. These pathogenic states influence each other, leading to persistent hyperglycemia and further initiation of and progression to T2D [12,13,14]. 

Accumulating evidence has highlighted a strong correlation between obesity, T2D, oxidative stress and mitochondrial dysfunction [15,16]. For instance, mitochondrial dysfunction in mature adipocytes has been linked to defects in fatty acid oxidation [17], dysregulation of glucose homeostasis oxidation [18] and secretion of adipokines [19]. Moreover, reduction in the oxidative capacity of brown adipocytes, together with impaired thermogenesis, has been linked to diet-induced obesity [20]. On the other hand, oxidative stress caused by hyperglycemia in diabetic patients can reduce insulin signaling, thus leading to insulin resistance [21]. Antioxidant mechanisms are diminished in these patients, which can further augment oxidative stress. Together, hyperglycemia and insulin resistance may also alter mitochondrial function, and insulin action can be impaired by cytokines in response to metabolic stress [22]. 

Recently, studies conducted both in human and animal models suggest that obesity and T2D are associated with alterations in the gut microbiota (known as dysbiosis) and related biological pathways [23]. Gut microbes seem to exert a crucial role in the development of metabolic diseases. Indeed, they can affect the host’s metabolic balance by modulating appetite, energy absorption, hepatic fatty storage, gut motility and lipid and glucose metabolisms [23,24]. Moreover, changes in gut microbiota homeostasis can increase intestinal permeability, thus promoting the translocation of bacterial endotoxins into the systemic circulation and further facilitating the metabolic endotoxemia and low-grade inflammation status that characterize these conditions [25]. Unfortunately, the specific mechanisms by which gut microbiota contribute to the pathogenesis of these metabolic disorders are not clearly understood. 

Interestingly, a connection between mitochondria and microbiota has been suggested over the last few years [26,27,28]. Gut microbiota have been shown to regulate crucial transcription factors, coactivators and enzymes implicated in mitochondrial biogenesis, metabolism and oxidative/nitrosative stress [26,27,28,29,30]. Moreover, the literature suggests that microbiota metabolites interfere with autophagosome formation, thus regulating the activation of the inflammasome and the production of inflammatory cytokines, both key players in these chronic metabolic disorders [26]. However, once again, the underlying processes require further study and clarification. 

Our aim is to provide an overview of the existing literature concerning the role of oxidative stress, mitochondrial dysfunction, inflammasome activation and gut dysbiosis in the onset of obesity and T2D. The review focuses on the fascinating inter-talk between mitochondrial function and intestinal microbiota and discusses the potential mechanisms by which they may become the key to therapeutic strategies to manage these metabolic diseases.

## 2. Mitochondrial Dysfunction in Obesity and T2D

### 2.1. Mitochondrial ROS

Reactive oxygen species (ROS) are oxygen-containing species able to react with or oxidize cytoplasmic molecules such as proteins, nucleic acids and lipids. They include hydroxyl radicals (OH•), superoxide anion (O^2-^) and hydrogen peroxide (H_2_O_2_), among others, that act as signaling mediators in many biological processes (cell development, differentiation and death) [31]. ROS are essentially produced by mitochondria, NADPH oxidase and nitric oxide (NO•) and are generated by aerobic metabolism or in response to cytokines or bacterial invasion, during which they act as cell defense modulators [32].

In this sense, growing evidence suggests that ROS are essential secondary messengers of several biological redox-signaling pathways. For instance, the moderate increase in ROS levels in lactate pretreated neuroblastoma cells provides resistance against cellular stress through the activation of the mTOR and PI3K pathways [33]. The metabolic shuttle between neurons and astrocytes depends on the NAD+/NADH redox equilibrium and oxidation of lactate and pyruvate [34,35]. In addition, the acute and controlled burst of H_2_O_2_ due to insulin stimulation results in the inhibition of tyrosine phosphatase activity, thus leading to an increase in tyrosine phosphorylation and enhancement of the insulin cascade [21]. Interestingly, studies have revealed that mitochondrial ROS generation via mTORC1-dependent protein translation modulates the induction of peroxisome proliferator-activated receptor (PPAR)-γ transcriptional machinery and triggers adipocyte differentiation signaling in human mesenchymal stem cells [36]. All this evidence affirms that ROS interacts with cellular redox-sensitive elements to shape and finely modulate downstream signaling events in a cell-specific and context-specific manner, thus ensuring oxidative cellular homeostasis. 

It is now widely accepted that, under physiological conditions, oxidative cellular homeostasis is achieved through a correct balance between the generation of ROS and the action of the antioxidant defense system in charge of their neutralization [37]. Specifically, these defenses include enzymatic or nonenzymatic systems such as superoxide dismutase (SOD), glutathione peroxidase (GPx), catalase or Kelch-like ECH-associated protein 1 (Keap1)-NRF2-ARE and are able to neutralize the reactivity of ROS-mediated cellular signaling and facilitate the anti-inflammatory response [38]. However, ROS production can be significantly enhanced in response to various stimuli, thus leading to an oxidative stress state. In this sense, an imbalance between the production and inactivation of ROS [38] promotes a direct or indirect cellular dysfunction, which may contribute to the onset of a variety of human diseases, including obesity and T2D [16].

### 2.2. Oxidative/Nitrosative Stress Signaling

As discussed above, oxidative stress, usually resulting from either excessive mitochondrial ROS production, creates the primary risk factor for the development of several diseases. In this way, mitochondria acquire a fundamental role in human health. In fact, they generate most of the cell’s supply energy. This process is mediated by electron transfer through the electron transport chain (ETC), which is composed of four different multiprotein complexes (I–IV) coupled to the ATP synthase, also named complex V [39]. Mitochondria perform key biochemical functions essential for metabolic homeostasis. First of all, they have an extensive communication system that connects them with the cell environment; for example, they communicate with the nucleus or cytoplasmic molecules to trigger signaling pathways like autophagy, reticulum stress and apoptosis [40]. Moreover, mitochondria respond to different stimuli or environmental stresses by modulating their morphology and distribution, as well as by promoting fission and fusion. Fusion alleviates stress by promoting the complementation between partially damaged mitochondria, while fission generates new organelles and removes the damaged ones, facilitating their apoptosis [41]. These mitochondrial dynamics allow the mitochondrion to adapt to intracellular signaling and contribute to cellular homeostasis. Unfortunately, mutations in these key components and defects in mitochondrial dynamics have been associated with numerous human diseases, including obesity and T2D. In this context, data reveal that mutations of Mitofusin 2 (Mfn2), a protein related to mitochondrial fusion, disrupt mitochondrial dynamics, which affects the motor neurons and muscle cells and leads to neurological and vascular diseases [42]. Bach et al. observed that higher levels of Mfn2 enhanced the glucose disposal rate in human skeletal muscles of obese and T2D subjects. Moreover, the BMI displayed an inversely proportional relationship [43], whereas chemical chaperones or the antioxidant N-acetylcysteine (NAC) ameliorated the glucose tolerance and insulin signaling in liver-specific Mfn2 knockout mice [44]. 

Since mitochondria are also vulnerable to oxidative stress, vicious cycles involving interactions between mitochondrial dysfunction and oxidative stress can contribute to the initiation and/or amplification of mitochondrial ROS. Mitochondrial ROS (mtROS) overproduction, mainly due to electron leakage from the ETC during oxidative phosphorylation, tilts the redox balance and activates cytosolic signaling pathways. In this sense, an increased nuclear gene expression of nuclear factor kappa-light-chain-enhancer of activated β cells NF-κB, catalase and NRF2/KEAP1 cascade signaling in murine models has been found to strengthen antioxidant cell defenses and restore balance [45,46]. It should be taken into account that there exist several antioxidant NF-κB targets that relieve the severity of the mitochondrial oxidative stress generated. Complex proteins like copper zinc superoxide dismutase, thioredoxins and ferritin heavy chains are clear examples of protective targets of mitochondria and cell survival [47]. 

In addition to mtROS, mitochondria can also generate reactive nitrogen species (RNS), which originate from NO. Interactions between NO and ROS result in peroxynitrite (ONOO-) formation and induce changes in the protein structure and/or activity via S-nitrosylation of the thiol groups and by nitrating irreversible tyrosine residues [48]. Exposure to NO in chronic situations induces mitochondrial oxygen consumption inhibition, whereas ONOO- overproduction can also inhibit complex I, complex II/III, cytochrome oxidase (complex IV) and ATP synthase of the mitochondrial ETC and increase the mitochondrial proton permeability [49,50]. These actions have important consequences for the redox state of the mitochondria, which eventually increases ROS production. Furthermore, it has recently been reported that NO exerts beneficial actions such as the improvement of cardiac contractility or pulmonary vascular tone, though it can also induce mitochondrial dysfunction and NF-κB activation in sepsis [51,52]. 

**Figure 1:**
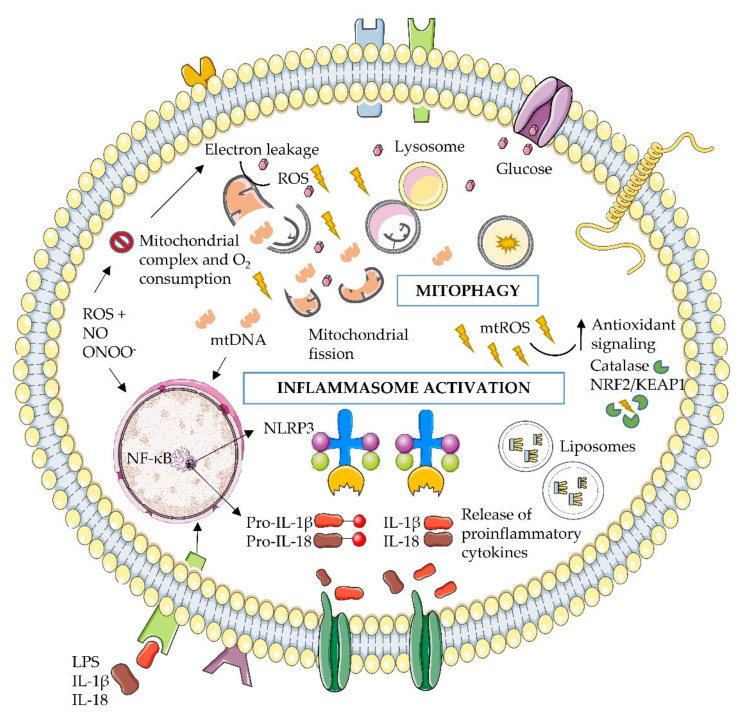
General mechanisms of ROS-induced mitochondrial dysfunction in obese and type 2 diabetes (T2D) patients. Dysregulated/impaired systemic inflammation, glucotoxicity and lipotoxicity triggers mtROS and RNS generation by mitochondrial ETC. Nitrosative stress also inhibits mitochondrial complexes and O_2_ consumption, as it is more reactive to ROS. As a result, the cytokines pro-IL-1β and pro-IL-18 and the inflammasome complex are activated through NF-кB translocation in the nucleus. Moreover, mtROS stimulates an increase in antioxidant defense signaling in order to modulate oxidative stress homeostasis. Disrupted mitochondria are targeted by autophagosomes in a process called mitophagy, which aims to restore the mitochondrial function. Abbreviations: ETC, electron transport chain; IL-1β, interleukin-1β; IL-18, interleukin-18; KEAP1, Kelch-like ECH-associated protein 1; mtROS, mitochondrial ROS; NF-кB, nuclear factor kappa-light-chain-enhancer of activated B cells; NRF2, nuclear factor erythroid 2-related factor 2; RNS, reactive nitrogen species; NO, nitric oxide; ONOO-, peroxynitrite; NLRP3, NLRP3 (nucleotide oligomerization domain (NOD), leucine-rich repeat (LRR) and pyrin domain (PYD)) and ROS, reactive oxygen species.

In this context, mitochondrial oxidative stress damage activates a selective recycling cascade with the aim of eliminating the dysfunctional mitochondrial section and restoring the redox equilibrium. This process is known as mitophagy and takes place through interactions between the mitochondrial membrane and cytosolic cascade signaling. Fission-induced mitochondrial depolarization and fragmentation tag this organelle to autophagosomes through PARKIN-PINK1 signaling [53]. Specifically, PINK1 accumulates on the surface of damaged mitochondria, where it rapidly recruits and activates Parkin’s E3 ubiquitin ligase activity. Thus, dysfunctional mitochondria are engulfed within autophagosomes and then degraded by lysosomes [53]. In addition to PARKIN-PINK1, other key effectors in the autophagy pathway, such as BCL2 and the adenovirus E1B 19-kDa-interacting protein 3 (BNIP3) and BNIP3-like (BNIP3L, also known as NIX), contribute to the clearance of mitochondria. Specifically, these proteins bring mitochondria to autophagosomes through direct interactions with microtubule-associated protein 1A/1B-light chain 3 (LC3), a ubiquitin-like modifier that is required for the growth of autophagosomes [54]. The depletion of Beclin 1 (autophagosome formation marker) or LC3B results in the accumulation of altered mitochondria and the cytosolic translocation of mitochondrial DNA (mtDNA) in murine models [55]. The suppression of mitophagy leads to the accumulation of dysfunctional ROS-generating mitochondria, and this, in turn, activates the NLRP3 (nucleotide oligomerization domain (NOD), leucine-rich repeat (LRR) and pyrin domain (PYD)) inflammasome, a cytosolic multiprotein complex that promotes inflammation, resulting in the release of proinflammatory IL-1β in response to pathogens and cellular distress. These inflammasome components are ubiquitinated and subsequently degraded by autophagy, as is pro-IL-1β [56]. Hence, there is an interplay between mitophagy and inflammasome proteins that controls the inflammasome activation, which occurs when autophagy-deficient cells accumulate abnormal mitochondrion complexes with increased mtROS and reduced mitochondrial membrane potential [57] (Figure 1). 

### 2.3. Mitochondrial Dysfunction in Obesity and T2D

Considered together, the above-mentioned knowledge shows that supraphysiological concentrations of ROS are responsible for metabolic and redox damage in cell homeostasis. In this sense, a substantial body of the literature is in agreement about the chronic and disruptive oxidative stress damage generated in obesity and T2D [15,16]. Firstly, in obese patients, the excess of macronutrients in adipose tissues stimulates them to release inflammatory mediators that, in turn, propagate the systemic inflammation associated with the development of hyperinsulinemia, insulin resistance and other comorbidities. Interestingly, the altered adipokine profile, which involves the upregulated expression and secretion of proinflammatory cytokines, contributes to the formation of toxic ROS and the subsequent generation of oxidative stress [58] through different processes. For example, in adipose tissue, obesity can induce oxidative stress mainly via catalytic activity of the nicotinamide adenine dinucleotide phosphate (NADPH) oxidase enzyme (NOX). Data reveal that, in the early and intermediate stages of obesity, NADPH oxidase-derived ROS are responsible for the recruitment of macrophages to the inflamed tissue, while in the later stages, mitochondrial-derived ROS maintain a proinflammatory environment and contribute to the development of insulin resistance [16,59]. Moreover, elevated lipid levels, which are abundant in obesity, seem to impair mitochondrial ETC and generate ROS, which can modify the chemical composition of lipids and makes them more reactive, thus maintaining a low level of inflammation [60]. It is also widely established that imbalanced levels of ROS are associated with adipogenesis dysfunction and adipocyte hypertrophy in adipose tissue and harmful systemic effects on vascular dysfunction, such as endothelial inflammation and hypercontractility [61,62]. 

In addition, oxidative stress has been closely related to β-cell dysfunction, one of the key players in the pathophysiology of T2D [60,61]. Pathological levels of ROS can activate NOX2 and the stress-sensitive serine/threonine kinase (JNK) in β cells and attenuate the insulin cascade, thus increasing insulin resistance [60,61]. Notably, β cells present very low levels of antioxidant enzymes, which make them more susceptible to oxidative stress [62]. This makes β cells highly sensitive to ROS-related signaling and susceptible to oxidative damage. Results by Sasaki et al. [63] reveal a key mechanism by which ROS impairs β-cell functions in diabetic rats: the activation of hypoxia-inducible factor 1α (Hif1α) results in remodeling towards increased aerobic glycolysis and suppressed cytokine-induced cell death. As a result, glucose oxidation and insulin secretion are impaired [63]. 

Growing evidence suggests that mitochondrial dysfunction and the excess generation of mtROS are also associated with the initiation of inflammation and development of insulin resistance [64]. Indeed, as previously reported, obesity and T2D are characterized by a cluster of metabolic factors that provoke increased reactive species production (ROS, NO and ONOO-) and mitochondrial DNA (mtDNA) synthesis, a higher mitochondrial fission and membrane depolarization [64]. This metabolic situation activates the NF-κB and, in turn, the synthesis and assembly of the inflammasome complex NLRP3 [65] (Figure 1). In particular, ROS can directly stimulate inflammasome assembly or be indirectly detected through cytoplasmic proteins that regulate and modulate the complicated activity of the inflammasome. Data reveal that ATP-mediated ROS generation can activate the PI3K pathway, and pharmacological inhibition of PI3K inhibits ATP-mediated caspase-1 activation, thereby pointing to PI3K as a key player in inflammasome activation downstream from ROS [66]. Similarly, research reveals that ROS can liberate TXNIP (thioredoxin-interacting protein), a protein linked to insulin resistance, which then interacts with NLRP3 and stimulates inflammasome assembly [67]. As a result, the inflammasome complex induces procaspase-1 activation, which is followed by IL-1β and IL-18 maturation (Figure 1). Thus, the release of these proinflammatory mediators into the systemic circulation leads to the initiation and/or promoting of a state of chronic inflammation that characterizes obesity and T2D [64]. Of note, under physiologically stressed conditions, regulation of the cellular inflammatory state and redox homeostasis occurs primarily at the transcriptional level, and the Nrf2/Keap1/ARE pathway (nuclear factor E2-related factor 2/Kelch-like ECH-associated protein 1/antioxidant response element) is the primary mediator of this response [68]. Cysteine residues on Keap1 are modified, resulting in the translocation and stabilization of Nrf2 into the nucleus, where it moves closer and binds to the promoter region of the ARE. Thus, Nrf2-ARE initiates the transcription of various cytoprotective enzymes that facilitate cellular survival through different processes, including inflammatory inhibition, the transport of toxic metabolites and the upregulation of antioxidant function [68]. However, there is accumulating evidence to associate this pathway with diabetic dysfunction in various cell types and tissues [69,70]. Indeed, the Nrf2/Keap1 pathway is shown to be dysregulated and functionally insufficient in diabetic animal models [70]. The same study revealed that the forced upregulation of Nrf2-directed transcription through the knockdown of Keap1 restores redox and inflammatory homeostasis [70]. These results endorse a mutual contribution of a systemic oxidative overload and an impaired Nrf2 antioxidant signaling pathway to the inflammatory state in diabetes. 

In light of the above, it seems clear that obesity and T2D are associated with chronically increased levels of oxidative stress, which, in turn, can trigger mitochondrial dysfunction, exacerbate the inflammatory process and lead to insulin resistance. It thus seems entirely possible that this oxidative stress and mitochondrial dysfunction is the molecular tipping point in the progression of poor health associated with these metabolic diseases. Importantly, a clear idea of the role of oxidative stress and mitochondrial dysfunction in these processes would allow the identification of potential therapeutic targets to slow or prevent the related health problems of obese and diabetic subjects. 

## 3. Gut Microbiota: A Novel Key Player in Obesity and T2D

The human gastrointestinal tract is colonized by large numbers of symbiotic, commensal and pathogenic microorganisms, including archaea, protozoa, fungi, viruses and bacteria, collectively identified as the “gut microbiota”. It consists of up to 100 trillion microbes, more than 1000 different bacteria species and five phyla (*Bacteroidetes*, *Firmicutes*, *Proteobacteria*, *Actinobacteria* and *Verrucomicrobia*), of which two are dominant: *Bacteroidetes* (*Bacteroides*, *Xylanibacter* and *Prevotella*) and *Firmicutes* (*Clostridium*, *Ruminococcus*, *Eubacterium*, *Lactobacillus*, *Roseburia* and *Faecalibacterium*) [71]. The gut microbiota serves several essential functions: preventing colonization by pathogens; producing vitamins such as vitamin K, folate and biotin; participating in energy regulation; fermenting dietary fibers in short-chain fatty acids (SCFAs); metabolizing xenobiotics; modulating brain activity by interacting with the enteric nervous system and assisting in the development of a mature immune system [71]. The bacterial quantity and diversity progressively increase from the stomach to the colon [72], with the colon being home of the densest and metabolically most active community. 

Different studies have assumed that the first contact with colonizing bacteria occurs in the birth canal. In this regard, distinctive microbiomes have been found in the placenta, umbilical cord and amniotic cavity; such microorganisms belong to the *Bacteroidetes*, *Firmicutes*, *Fusobacteria*, *Tenericutes* and *Proteobacteria* phyla; are not pathogenic and constitute the initial bacteria that colonize the fetal gastrointestinal tract [73,74]. In the first days of life, different types of bacteria, mainly aerobic microorganisms, rapidly colonize the gut, reaching its maximum density within 72 h of birth. This microbiota composition in the infant depends on the type of delivery; birth gestational date; methods of milk feeding; weaning period and external factors such as illness, the use of antibiotics and changes in diet [75]. The phylogenetic diversity of microbiota gradually increases and favors colonization by anaerobic bacteria [76]. Three years after birth, the gut microbiota has progressed to a “mature” stage, which remains essentially stable throughout adulthood [76,77]. Although there is not a unique optimal composition of the gut’s microorganisms, a healthy host-microbiota balance must be maintained in order to guarantee immune and metabolic functions and prevent disease development as much as possible. Unfortunately, changes in intestinal microbiota and in microbial metabolite composition, due mainly to an “unhealthy” diet, lifestyle factors, the use of antibiotics [78] and emotional and physiological stress [79], may affect the gut homeostasis and lead to dysregulation of the microbiota composition (also known as dysbiosis), as well as nonspecific inflammation and diseases [80].

**Figure 2:**
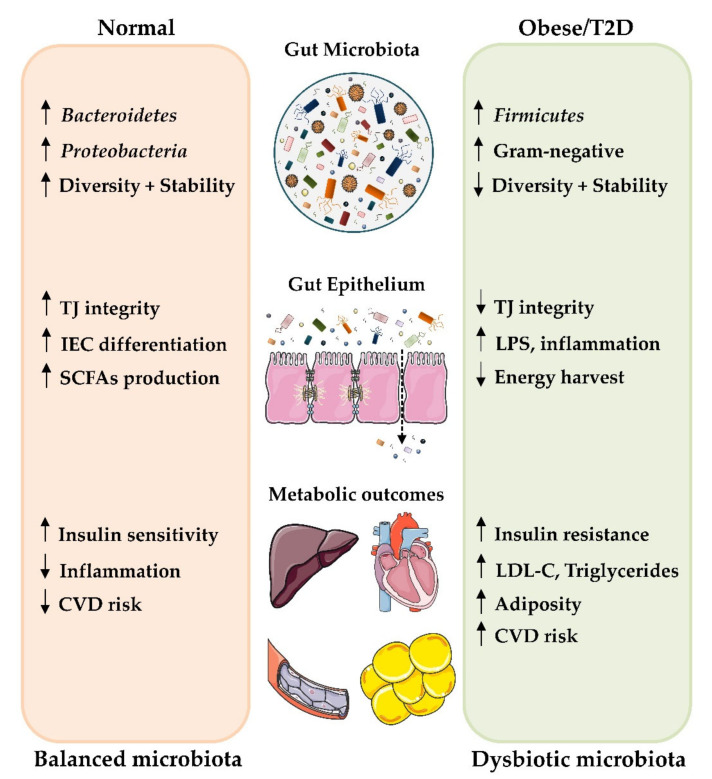
Role of gut microbiota in the development of obesity and T2D, including some of the mechanisms thought to contribute to alterations of the host metabolic state. Up and down arrows indicate an increase and decrease, respectively. Abbreviations: IEC, intestinal epithelial cells; LPS, lipopolysaccharide; LDL-C, low-density lipoproteins-cholesterol; SCFAs, short-chain fatty acids; CVD, cardiovascular disease and TJ, tight junction.

Numerous studies in animal models and humans have demonstrated that gut microbiota dysbiosis can be considered a major player in the development of obesity and T2D [81,82,83]. Specific bacterial phyla, class, order or species and/or bacterial activity seems to be detrimental or beneficial to the onset of such syndromes. Indeed, although the composition of the intestinal microbiota fluctuates markedly in healthy individuals, those exhibiting insulin resistance, overall adiposity and dyslipidemia are characterized by low bacterial variety [84] (Figure 2).

Obese and T2D individuals report consistent changes in the intestinal microbiota composition in comparison to lean individuals. Of note, the prevalence of *Bacteroidetes* is lower in obese and diabetic subjects and increases with weight loss [85,86]. *Clostridium* and *Lactobacillus* species are correlated with insulin resistance; *Clostridium* is negatively associated with fasting glucose and glycated hemoglobin levels, while *Lactobacillus* is positively correlated with such parameters [87]. In addition, two large metagenome-wide association studies showed that T2D individuals present a lower proportion of butyrate-producing *Clostridiales* (*Faecalibacterium prausnitzii* and *Roseburia*) and higher proportions of *Clostridiales* that do not produce butyrate, indicating a protective role of butyrate-producing bacteria against T2D [81,87]. In addition, several reports show that obese people present a low amount of *Akkermansia muciniphila*, a mucin-degrading bacterium responsible for the unsealing of the intestinal barrier [88]. A lower abundance of this microorganism leads to an increased gut permeability and a further translocation of bacterial endotoxins into the systemic circulation, thus contributing to the chronic inflammation related to both obesity and insulin resistance [89]. 

**Figure 3:**
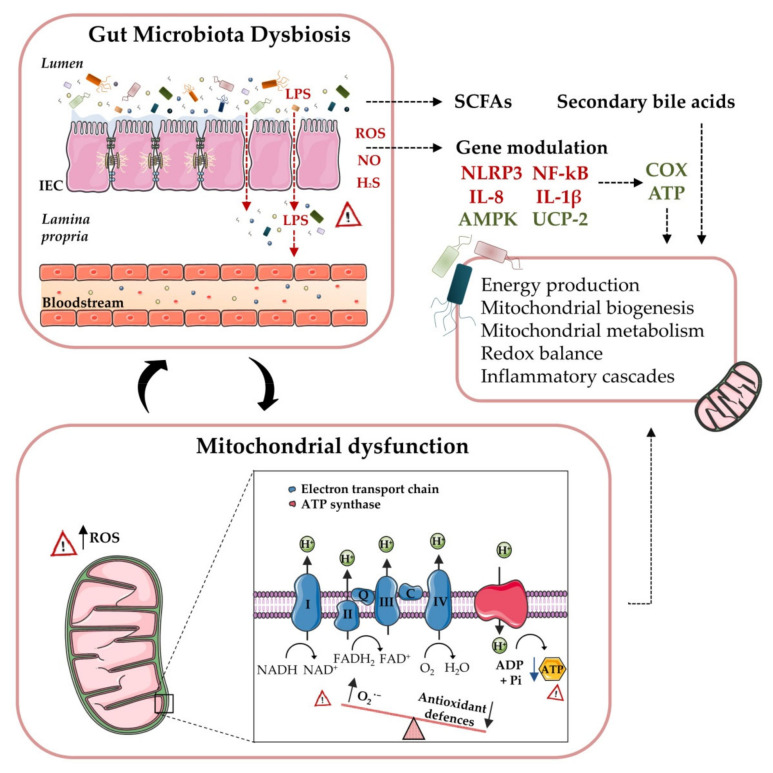
Microbiota-mitochondria inter-talk: Increased gut permeability in obese and T2D patients can induce LPS translocation to the bloodstream, along with a further inflammatory cascade, ROS and H_2_S production, as well as inflammasome NLRP3 activation, thus resulting in a mitochondria-mediated inflammatory response. The inhibition of AMPK activation and decreased expression of UCP-2 lead to a reduced glucose metabolism and to the partial uncoupling of mitochondrial oxidative phosphorylation, as well as an increased ROS production. Moreover, microbiota-released metabolites can also undermine or promote mitochondrial biogenesis and energy metabolism. Concurrently, mitochondrial ROS production can regulate gut microbiota activity and composition by modulating mucosal immune responses and intestinal barrier functions. Abbreviations: AMPK, AMP-activated protein kinase; ATP, adenosine triphosphate; COX, cyclooxygenase; H_2_S, hydrogen sulphide; IEC, intestinal epithelial cells; LPS, lipopolysaccharide; NLRP3, inflammasome NOD-like receptor family and pyrin domain containing 3; NF-κB, nuclear factor kappa-light-chain-enhancer of activated B cells; NO, nitrogen oxide; ROS, reactive oxygen species; SCFAs, short-chain fatty acids and UCP-2, uncoupling protein 2.

Unfortunately, the mechanisms by which gut microbiota contribute to the pathogenesis of these metabolic disorders are not clearly understood. In this sense, recent data have shed light on interactions between microbiota quality and diversity and mitochondrial function [26]. Of note, the intestinal microbiota seems to play a crucial role in the regulation of several transcription factors, transcriptional coactivators and enzymes associated with the mitochondrial biogenesis and metabolism [26,27,28,29,30]. Furthermore, microbiota metabolites seem to interfere with mitochondrial oxidative/nitrosative stress and autophagosome formation, thus regulating the activation of the NLRP3 inflammasome and the release of different inflammatory cytokines [26,28], all key players in chronic metabolic disorders (Figure 3). 

In the following sections, we will focus on the association between intestinal microbiota and mitochondrial function and discuss potential mechanisms by which they can be used as therapeutic strategies in obesity and T2D management.

## 4. Microbiota and Mitochondria in Obesity and T2D: Friends or Foes?

Recent evidence in microbiology has demonstrated that, despite their different roles, mitochondria and microbiota bacteria share many common features. Indeed, the endosymbiotic theory broadly states that the ancestor of mitochondria is a member of the alpha-proteobacteria phylum that developed a symbiotic relationship with the eukaryotic cell [90], thus giving it similar structural and functional features to bacteria. Both mitochondrial and bacterial membranes are degraded through similar autophagic systems; mitochondrial and bacterial ribosomes are more related to each other than either is to eukaryotic ribosomes and are both sensitive to antibiotics [91]. Some bacterial proteins can be brought into the host mitochondria because of the similarity of the mitochondrial and bacterial cytoplasmic protein targeting sequences [28].

Due to their essential roles in determining metabolic health, both the gut bacteria and mitochondria have become key targets in medical and biological investigations, potentially opening up new avenues for the treatment of obesity and T2D. The interaction between microbiota and mitochondria seems to occur principally through endocrine, immune and humoral links [92]. Notably, a role has been assigned to SCFAs, ROS, NO and hydrogen sulfide (H_2_S) in the cross-talk between the microbiota and mitochondria (Figure 3). This modulation depends on the diversity and quality of the gut bacteria to increase its pathogenic versus beneficial effects. Furthermore, the microbiota can directly interact with the host cell’s gene expression by promoting bacterial and mitochondrial DNA insertions in the nuclear genome [93]. 

## 5. SCFAs, Secondary Bile Acids and Mitochondria

One of the primary reasons for the rise of obesity and diabetes is dietary changes. In fact, the increased consumption of processed carbohydrates and inadequate amounts of dietary fibers have been recognized as significant risk factors for these metabolic diseases. 

Principle dietary fibers include soluble fibers such as inulin, pectin, hemicellulose and arabinoxylan and insoluble fibers such as cellulose. Soluble fibers are preferentially fermented by microbiota in the colon to produce SCFAs, saturated aliphatic organic acids that consist of one to six carbons, of which acetate (C2), propionate (C3) and butyrate (C4) are the most abundant (≥95%) [94]. Starches are difficult to degrade by digestive enzymes due to their structures, reach the colon where they are fermented and increase the SCFAs levels. Both soluble and insoluble fibers play essential roles at the intestinal level by ameliorating oxidative status and mucosal inflammation and regulating transepithelial fluid transport, as well as reinforcing the epithelial defense barrier [95]. However, the effects of SCFA are not restricted to the intestine, as they have been reported to enter the peripheral circulation and to cooperate in peripheral tissue metabolism. In systemic circulation, they influence the energy metabolism and homeostasis by regulating the mitochondrial functions and dynamics in the liver, brown adipocytes and skeletal muscle via the G protein-coupled receptor and FFA receptor (FFAR) signaling [96,97]. 

For instance, acetate, the most abundant SCFA, secreted by the phylum *Bacteroides*, can be used by the mitochondria as a source of energy [98], while butyrate, produced by *Firmicutes*, stimulates mitochondrial biogenesis via the inhibition of histone deacetylases [99], thus leading to increased energy expenditure and weight loss. Notably, Mollica et al. showed that sodium butyrate reduced ROS production and ameliorated mitochondrial function in the livers of insulin-resistant obese mice [100]. Similarly, Shuxian et al. revealed that both acetate and butyrate can prevent mitochondrial dysfunction and reduce oxidative and nitrosative stresses in human islets and β cells after exposure to the apoptosis inducer and metabolic stressor streptozotocin (STZ) [101]. In addition to these rescuing effects, SCFAs prevented the downregulation of the mitochondrial fusion genes Mfn1, Mfn2 and Opa1 and the upregulation of the fission genes Drp1 and Fis1 during STZ exposure. Acetate revealed more efficiency in inhibiting the ROS and increasing the metabolism, while butyrate had a weaker effect but was stronger in inhibiting NO generation and the SCFA receptor GPR41 [101]. 

Additionally, acetate and N-butyrate may also influence the metabolism and mitochondrial function in colonocytes through the activation of AMP-activated protein kinase (AMPK) [102,103], a crucial energy sensor able to regulate mitochondrial oxidative phosphorylation (OXPHOS) [104]. Similarly, SCFAs can activate the uncoupling protein 2 (UCP-2)-AMPK-acetyl-CoA carboxylase (ACC) pathway, which, in turn, leads to a downregulation of PPAR-γ gene expression, along with a reduction in lipogenesis and an increase in the AMP:ATP ratio [103]. This increased ratio may also stimulate AMPK in the liver, muscle [105] and adipose tissue [106], thus activating glucose uptake and mitochondria OXPHOS and decreasing lipid and protein synthesis.

Besides SCFA, the gut microbiota produces secondary bile acids that might influence the mitochondrial energy metabolism and biogenesis, making it a potential therapeutic target for endurance. The anaerobic bacteria of the genera *Eubacterium*, *Clostridium* and *Bacteroides* degrade 5–10% of the primary bile acids to produce secondary bile acids [107]. These secondary bile acids interact with mitochondria by regulating transcription factors associated with carbohydrates and the lipid metabolism, including G-coupled membrane protein 5 (TGR5) and farnesoid X receptor (FXR) [108]. While TGR5 induces ERK/DRP1-dependent mitochondrial fission and beige remodeling of white adipose tissue [109], FXR is a target of NAD-dependent protein deacetylase sirtuin-1 (SIRT1) [110], which regulates the carbohydrate response element binding protein (ChREBP), steroid response element binding protein-1c (SREBP-1c) and PPAR-α, thus stimulating fatty acid uptake and oxidation [111]. Growing evidence suggests that the secondary bile acid metabolism might also directly modify SIRT1 and the Fasting-induced adipose factor (FIAF) expression, as well as the intestinal barrier function, mitochondrial biogenesis and inflammation in different types of cells [112,113,114,115,116], thus indicating potential mechanisms by which microbiota and mitochondria can be used as therapeutic strategies in the management of obesity and T2D.

The majority of the above-mentioned reports are the results of research in animal models, while evidence obtained in humans is scarce. Therefore, it is necessary to further elucidate the potential mechanisms by which SCFAs and secondary bile acids produced by the gut microbiota regulate the metabolism of mitochondrial energy. In this sense, further research is warranted to clarify whether the manipulation of microbiota metabolites and related factors could provide more health-related benefits than modulating the quality and diversity of the microbiota. 

## 6. Microbiota, Mitochondria and the Immune System

Accumulating data suggest that interactions between the immunity and metabolism reveal a central role in the development of obesity-related chronic comorbidities. The impairment of both innate and adaptive immune systems leads to an enhanced risk of chronic low-grade inflammation, which, in turn, can cause metabolic dysfunction and chronic diseases, such as insulin resistance and T2D [117]. The gut microbiota has appeared as a critical factor in the early events that trigger the inflammation linked to obesity and metabolic dysfunction [23]. LPS, a major component of the Gram-negative bacterial outer membrane, binds to Toll-like receptors (mainly Toll-Like receptor 4, TLR4). TLRs are well-described immune transmembrane proteins able to activate NF-κB; upregulate inflammatory chemokines and cytokines and engage intracellular signaling pathways to control the nature, duration and magnitude of the inflammatory response [118]. As a result, and in response to microbial and inflammatory stimuli, phagocytes release ROS, either within the mitochondria or through a process termed “oxidative burst” via the NADPH oxidase complex. Simultaneously, an increase of intestinal permeability along with a subsequent translocation of immunogenetic bacterial products leads to the exacerbation of the inflammatory tone [119]. 

Although there are few data regarding the microbiota-mitochondria inter-talk in the immune system, studies in animal models revealed that LPS injections in cats determined a 40% decrease of cyclooxygenase (COX) activity, as well as a partial uncoupling of mitochondrial OXPHOS [120]. However, in another study, mice showed a decrease in the expression of UCP-2 and higher ROS production [26,93]. In 2014, Lee and Hüttemann proposed that LPS/TLR bindings stimulate the production of TNF-α and IL-6, thus activating tyrosine kinase and leading to downstream COX phosphorylation and impaired ATP production in mitochondria [121].

At the same time, the mitochondrial function seems to modify the intestinal microbiota composition and activity, since it is able to stimulate an immune response [122] when cellular damage and infectious microorganisms are detected. Similarly, immune cells are under the influence of the intestinal microbiota, whose supporting role in mitochondria functions and activities is becoming increasingly obvious.

These data suggest that the interaction between the innate immune system and the intestinal microbiota collectively contributes to the regulation of some mitochondrial oxidative functions and the further development of metabolic syndromes. However, the underlying mechanisms are still poorly understood, and further studies are needed to clarify them. 

## 7. Microbiota and Mitochondria: Potential Therapeutic Strategies in Obesity and T2D 

To achieve an adequate long-term metabolic control in obesity and T2D, a combination of pharmacological treatment and lifestyle changes is usually necessary. Due to a lack of efficacy and poor tolerability of the drugs currently in use [123], along with their high cost, research into new approaches is vital. In this sense, targeting microbiota may represent a new avenue for therapeutic approach to treat or prevent obesity, T2D and related metabolic disorders. These strategies include dietary manipulation, such as the use of probiotics, prebiotics or symbiotic, as well as the transplantation of fecal microbial communities. 

In addition, experimental studies and human clinical trials have shown the effects of various prebiotic and probiotic strains and their potential efficacy in ameliorating obesity, T2D and their related metabolic comorbidities. In particular, they seem to confer beneficial effects by reversing dysbiosis and restoring the gut functional integrity [124,125]. Similarly, *Lactobacillus acidophilus*, *Lactobacillus bulgaricus*, *Streptococcus thermophilus* and/or *Bifidobacterium lactis* administered for six to 12 weeks has been shown to be effective in improving glycemic control in adults with T2D [126]. In addition, the anti-obesogenic effects of symbiotic, prebiotic and probiotic compounds improve the glucose, lipid and carbohydrate metabolisms; insulin sensitivity; fasting blood glucose and antioxidant status, as well as reducing the metabolic stress in such patients [125,127]. For example, *B. animalis* subsp. *lactis* BB-12 and *L. acidophilus* La-5 administration in T2D patients reduces LDL-C, TC and hemoglobin A1c levels and increases GPx and erythrocyte SOD activity and the total antioxidant status with respect to the controls [128,129]. Although further studies are required to understand their underlying mechanisms of actions, other potential bacterial candidates, such as *Enterobacter halii*, *Akkermansia muciniphila* and *Saccharomyces cerevisiae* var. *boulardii*, have been identified as novel therapeutic strategies in the management of metabolic diseases [130]. 

Growing evidence suggests that fecal microbiota transplantation (FMT) is a valuable and promising therapeutic option for diabetes and obesity disorders [131]. FMT showed beneficial effects by attenuating the pancreatic islet β-cell destruction and improving the insulin resistance in T2D patients [131,132], as well as reducing the low-grade chronic inflammation caused by a microbiota imbalance [131]. Moreover, a recent study by Who et al. showed that altered gut microbiota mediates some of the antidiabetic effects of metformin, a drug widely used in T2D that inhibits complex I and enhances the ADP:ATP ratio, thereby activating liver AMPK to slow liver gluconeogenesis [133]. In this sense, the transfer of metformin-treated human microbiota (obtained before and four months after treatment) to germ-free mice improved the glucose tolerance and insulin sensitivity in mice receiving metformin-altered microbiota [134].

Although there are numerous studies about the function of FMT and its beneficial effects, the mechanism underlying the FMT-induced alleviation of such diseases remains to be determined, for which further controlled and randomized trials are required. On the other hand, assuming a central role of mitochondrial dysfunction in the development of obesity and T2D, novel therapeutic approaches have recently been developed to regulate and/or restore mitochondrial biogenesis, metabolism, respiration and function. Firstly, lifestyle modifications, such as diet and exercise, can offer several benefits, including an increased electron-transport activity, stimulation of mitochondrial biogenesis and dynamics, the activation of AMPK and the phosphorylation of the peroxisome proliferator-activated receptor-γ coactivator (PGC-1α), along with an improvement of insulin sensitivity [135,136]. 

In addition to nonpharmacological strategies, there are several pharmacological approaches to the treatment of mitochondrial dysfunction. In this regard, small molecules have been shown to modulate ROS production and, consequently, to ameliorate mitochondrial function. Drugs can act indirectly on the mitochondria by binding to regulatory targets in the cytosol or nucleus or directly through conjugation to lipophilic cations or to peptides, thus facilitating the drug effectiveness, limiting side effects and accelerating the delivery [137,138]. These different molecules include succinate, antioxidants, vitamin B1, vitamin E, NAC, coenzyme Q, α-lipoic acid, proteins and substrates of the ETC [139,140]. 

One of the most widely known mitochondria-targeting substances is mitoquinone (MitoQ), an antioxidant compound incorporated into the matrix-facing surface of the inner mitochondrial membrane (IMM) that decreases the ROS and modulates the antioxidant activities, such as GPx1, under oxidative stress conditions. In this sense, recent data have shown the positive effects of MitoQ in leukocytes from T2D patients [141]; the findings revealed that MitoQ has an antioxidant and anti-inflammatory action by reducing ROS production, TNF-α and leukocyte-endothelium interactions through a reduction of NF-κB [141]. Moreover, Pung YF et al. demonstrated that MitoQ normalized the metabolic profile in obese rats, decreasing the lipid peroxidation and attenuating the levels of the UCP-2 protein to values like those in lean animals [142]. Interestingly, a recent in vitro study demonstrated the potential benefits of MitoQ for pancreatic β-cell functions by modulating the mitochondrial function and ameliorating the NF-κB activation and endoplasmic reticulum ER stress [143].

Another group of mitochondria-targeted antioxidants includes water-soluble Szeto Schiller (SS) peptides, small cell permeable antioxidants that are selectively and rapidly taken up into the IMM of different types of cells, including renal, embryonic and endothelial cells. It has been shown that they can modulate mitochondrial ROS production and reduce the mitochondrial permeability transition, preventing necrosis and apoptosis, which are promoted by inhibition of the mitochondrial ETC or oxidative stress [144]. Another current strategy under evaluation for mitochondrial targeting is that of liposomes, of interest due to their clearance rates after systemic injection. Liposomes are self-assembling colloidal structures mainly composed of phosphatidylglycerol, cholesterol and phosphatidylcholine. In addition, several studies suggest that mitochondria-targeting liposomes are able to enhance the drug efficacy in both in vitro and in vivo models by delivering therapeutic moieties to the mitochondria [144].

It is clear that, due to the mitochondrial bilayer structure and their negative potential natures, therapeutic drugs have recurring problems in reaching mitochondria. To overcome this difficulty, researchers have developed different pharmaceutical preparations, such as polymeric nanoparticles, inorganic nanoparticles, liposomes and those modified by mitochondriotropic moieties that allow specific targeting. Although they have been extensively proved as an effective therapy for diabetes and its related comorbidities, more investigation is needed to confirm their use as a viable therapeutic option.

In summary, the mitochondria and microbiota are key targets in the treatment of obesity and T2D. Different compounds and strategies have been developed to alleviate mitochondrial dysfunction and gut dysbiosis in these conditions. Various targets and mechanisms of action have been focused on, such as the regulation of mitochondrial dynamics, stimulation of mitochondrial biogenesis, regulation of apoptosis and oxidative stress and restoration of gut homeostasis and intestinal barrier integrity. Accumulated evidence suggests that therapy that targets mitochondria-microbiota inter-talk may constitute novel ways to treat these diseases or to minimize their complications, since it would allow a wider range of action, rather than interfering with the mitochondria and microbiota separately. Although recent data have thrown light on the intricate molecular signaling between intestinal microbiome and mitochondria in the pathogenesis of obesity and T2D, further in-depth studies are required.

## 8. Concluding Remarks and Future Perspectives

The prevalence of obesity and T2D has resulted in pandemic levels worldwide, and their associated comorbidities have increased the risk of morbidity and mortality in the last decade. Emerging evidence suggests that mitochondrial dysfunction and oxidative stress are closely related to these disorders, positioning themselves as critical determinants of the autophagic process, endoplasmic reticulum stress, insulin sensitivity and inflammation. Moreover, reported data have highlighted the key role of the gut microbiota in the pathogenesis of these metabolic disorders and their associated cardiovascular complications. Indeed, gut dysbiosis has been linked to weight gain, mucosal immune response and inflammatory damage, as well as insulin resistance and glucose metabolism, through the activation of multiple metabolic pathways, including lipopolysaccharide, short-chain fatty acids, aromatic amino acids and their related metabolites. 

In recent years, a prominent role has been demonstrated for gut microbiota in the modulation of mitochondrial functions and vice versa. Indeed, it would appear that microbes are able to influence the mitochondrial energy biogenesis and metabolism, alter the epithelial barrier function, induce inflammasome signaling and activate immune cells. Concurrently, mitochondrial ROS production can regulate the gut microbiota activity and composition by modulating mucosal immune responses and intestinal barrier functions. Considering that mitochondria derived from an ancestral bacterial endosymbiosis, it is not surprising that a special connection exists between this organelle and the bacteria. The scientific data reported in this review suggest that microbiota-mitochondria inter-talk represents a promising avenue for exploring novel treatment targets for obesity and T2D. These precise therapeutic interventions could modulate imbalances in the composition of gut microbiota, as well as the alterations in mitochondrial biogenesis, metabolism and oxidative/nitrosative stress that define such diseases. 

Although the knowledge of this inter-talk has increased considerably, many fundamental issues related to the topic of this review remain unsolved at present. For example: “Which primary species of gut microbiota specifically interfere with mitochondrial function?”, “Is ROS production directly correlated to microbiome species diversity rather than microbiota metabolites?”, “Which specific molecular triggers govern the mitochondria-microbiota interplay?” and “What are the possible factors/pathways that would allow their use as a new therapeutic approach in obesity and T2D?” 

Considering these questions, further research on microbiota-mitochondria inter-talk is obviously required in order to examine and clarify the mechanisms that could be used as potential strategies in obesity and T2D management.

## Survey Methodology

A literature search aimed at compiling any published data about the key roles of mitochondrial dysfunction and gut dysbiosis in the onset of obesity and T2D, with a special focus on microbiota-mitochondria inter-talk. We searched for literature relevant to the topic of the article by using databases such as PubMed and Google Scholar. The keywords oxidative stress, mitochondrial dysfunction, inflammasome activation, gut dysbiosis, microbiota-mitochondria inter-talk, obesity and type 2 diabetes were used to search. Screened articles were used as references for this review.
